# Serological Survey of Aujeszky’s Disease in Wild Boar from Southeastern France

**DOI:** 10.3390/pathogens11101107

**Published:** 2022-09-27

**Authors:** Younes Laidoudi, Bernard Davoust, Stéphanie Watier-Grillot, Aurélie Oger, Marie-Frédérique Le Potier, Céline Deblanc

**Affiliations:** 1 Aix Marseille Univ, IRD, AP-HM, MEPHI, 13005 Marseille, France; 2 IHU Méditerranée Infection, 13005 Marseille, France; 3 Animal Epidemiology Expert Group, French Military Health Service, 37076 Tours, France; 4 ANSES, Ploufragan-Plouzané-Niort Laboratory, Swine Virology Immunology Unit, National Reference Laboratory and OIE Reference Laboratory for Aujeszky’s Disease, 22440 Ploufragan, France

**Keywords:** anti-gB antibodies, Aujeszky’s disease virus, wild boar, seroprevalence, France

## Abstract

Aujeszky’s disease virus (ADV), also known as pseudorabies virus, causes an important neurological infection with a major economic and health impact on animal husbandry. Here, we serologically screened muscle fluid from wild boar (*Sus scrofa*) for the presence of anti-ADV antibodies. Animals were caught during two hunting seasons (2019–2020 and 2021–2022) from three areas in southeastern France known to be endemic with wild boar populations. A total of 30.33% of the 399 tested animals scored positive for anti-glycoprotein B antibodies directed against ADV using a commercial competitive ELISA test. A significant effect (*p*-value < 0.0001) of the geographical location and animal age on ADV seroprevalence was observed. The results of this study confirmed the importance of wild boar in the epidemiology of ADV in southeastern France.

## 1. Introduction

The earliest description of Aujeszky’s disease (AD) dates back more than two centuries. Based on the observed symptoms, the disease was first described as rabies-like or “pseudorabies” [1,2,3]. Thus, the disease was well recognized by its clinical picture, though the causative agent was unknown until 1902, when the Hungarian physician Aladár Aujeszky isolated the causative agent of pseudorabies disease from a diseased ox, a dog, and a cat [4]. Subsequently, pseudorabies disease became widely known as Aujeszky’s disease. 

The Aujeszky’s disease virus (ADV), also known as Suid Herpesvirus-1 or SuHV-1, belongs to the family *Herpesviridae*, subfamily *Alphaherpesvirinae*, genus *Varicellovirus*. The virus is a filtrable particle of 180 nm [5,6] known for its high neurotropism, with transneuronal transmission (direct neuron-to-neuron transmission) promoting neurological disorders in susceptible animals [7]. ADV can infect all mammals, except higher primates. However, only swine (i.e., pigs and wild boar) are considered the natural host, due to their ability to survive a productive infection [7]. The clinical signs of ADV infection in swine vary, largely depending on ADV strain and age and animal status, ranging from neurological disorders and death in young animals to respiratory and reproductive disorders in adults, which become latent carriers after recovery. However, it causes a range of clinical symptoms (i.e., pruritus, tremors, convulsions, incoordination, paralysis) leading to fatal outcomes in susceptible non-swine animals [7,8,9]. 

The implementation of the DIVA (differentiating infected from vaccinated animals) strategy, along with strict sanitary measures yielded control or even eradication of AD in pig farms in numerous countries [10]. However, despite the successful application of this eradication program, the continuous emergence of new outbreaks involving wild boar is still anticipated [11]. The presence of ADV in wild boar populations represents not only a risk of reintroduction into pig herds, but also a real threat to wild and domestic mammals [12,13,14,15]. In France, the complete eradication of AD was achieved in 2008 for all of mainland France. However, outdoor farms remain exposed to the infection due to the proximity of infected wild boar. On the other hand, the number of hunting dogs with fatal AD continuously increases [16]. The last ADV surveys carried out on wild boar from several French administrative departments revealed an infection rate of up to 54% [17,18]. In France, the verification of the AD-free status is based on serological surveillance in breeder-multiplier and outdoor swine farms, as well as event-based surveillance in all domestic mammals [19], but no surveillance is conducted on wild carcasses. In the absence of new updated epidemiological data from France, as in many other European countries, the control of AD remains difficult. To this end, the present study aimed to provide an update on the epidemiology of AD in wild boar from southeastern France. 

## 2. Results

Overall, 30.33% (CI: [25.82; 34.84]) of the 399 tested animals were scored positive by ELISA assay. The detailed results of seroprevalence are shown in Table 1. All animals that tested positive were from Solenzara and Canjuers camps, while none of the tested animals from the Carpiagne camp scored positive during both seasons. Age and geographical origin significantly affected AD seroprevalence. No statistical effect of gender was observed. 

## 3. Discussion

The present study reports non-negligeable seroprevalence of AD in wild boars and updates the information on its epidemiology in southeastern mainland France and Haute-Corse areas. As access to biological samples of wild boar is difficult, the present investigation was conducted on the available hunted animals, which may explain the significant difference between the investigated areas. In addition, samples analyzed were muscle exudates and not sera. Despite the low sensitivity (20 times lower) of this technique compared to sera sampling, as previously demonstrated on pig samples [20], it remains a useful technique for screening ADV from hunted wild boar [21]. In general, serological assays targeting anti-gB antibodies are recommended by the OIE Sanitary Code and Community Decision 2008/185/EC for the maintenance of AD-free status by the implementation of measures to prevent any transmission of the virus between wildlife and domestic animals (https://info.agriculture.gouv.fr/gedei/site/bo-agri/instruction-2016-452. Accessed on 20 September 2022). Other assays, such as virus isolation and genomic-based detection, can also be used to detect and/or confirm the infection [22]. However, the shortness of the period in which the virus is isolable decreases the sensitivity of viral-based detection and/or virus isolation from boar samples, as previously demonstrated by Müller et al [23]. 

No obvious clinical picture of AD was noticed for the studied animals. ADV strains from Europe and North America appear to be attenuated in adult pigs, although the disease was observed in very young piglets and clinical manifestation in adult wild suids occurs rarely [22]. 

The overall seroprevalence of 30.3% reported here from a wild boar population in southeastern France is comparable to a previous report of 45.1% seroprevalence observed in this area [18]. Overall, when present, the recorded seroprevalence range of 22.2–76.9% is comparable to that of recent European reports from Germany (12.09%, n = 108,748) [24], the Iberian peninsula (42.6%, n = 235) [23] and northwest Italy (9.98%, n = 902) [25]. These data reflect a strong circulation of ADV among the wild boar population in southeastern Europe. As a result, pig farms are particularly threatened, along with other domestic and wild mammals. For example, in 2018, an outbreak was reported in a southeastern farm located near a forest and was disseminated throughout another farm, where the origin of the ADV was the wild boar population (Ministère de l’agriculture et de la souveraineté alimentaire, 2019) [26]. Similarly, two other outbreaks in outdoor pig herds have been detected in 2021 and 2022 in the same region [27]. Southeastern France is not an area with a high density of pig farms, but the recurrent detection of outbreaks, and the results of the present study carried out in wild boars, clearly demonstrate that outdoor pig farms are particularly at risk in this area. Given the potential for direct and indirect contact with infected wild boar, the possibility of transmission of the disease to other animal species kept in the area (cattle, goats, sheep, etc.) should not be overlooked. In compliance with the new European regulatory framework on animal health (Reg. EU 2016/429) and the WOAH (World Organisation for Animal Health) recommendations, the vaccination of pigs against ADV infection is not permitted in countries with AD-free status [15,28]. Consequently, only biosecurity measures can prevent the spread of AD (Ministerial Order of 16 October 2018) [29]. Continued vigilance on the part of farmers and scheduled surveillance of all outdoor pig herds, therefore, remains a priority to maintain AD-free status.

Natural ADV infection in wild canids is not new. According to the literature, more than fourteen wild foxes [30,31,32], over 1200 captive foxes [33], three wolves [34], three captive coyotes [35], four brown [36] and one black bear [37] and six raccoons [38] have been reported from different countries. In all cases, severe signs with fatal outcomes were noted [7]. However, and despite the close predator-prey relationship between wild canids (wolves and foxes) and wild boar populations from the studied areas [39], there has been no report of ADV infection among these canids, according to hunters and foresters. This may be due to the predation behavior of wolves, which feed on wild boar offspring that are protected against ADV through maternal immunity. An experimental study by Müller et al. (2005) showed that wild boar offspring from an immunized sow remained protected for up to 27 weeks post-partum [40]. Accordingly, the present data showed that AD seroprevalence becoming significant with age is increasing. Domestic carnivores (dogs and cats) are also susceptible to ADV infection, with fatal outcomes [7]. For example, in France, several canine cases of ADV infection have occurred after wild boar hunting [41,42], as confirmed previously by genomic studies of French ADV canine strains [43]. Domestic canids are exposed to ADV infection through physical contact with an infected boar during hunting, or after eating contaminated raw meat or offal, as previously reported in Europe [44] and France [45]. In the present study, none of the hunting dogs used to catch wild boar was diseased. This may be due to knowledge of AD transmission by the hunters, who avoid feeding their dogs with raw meat and offal of the wild boar. However, despite this precaution, exposure risk to hunting dogs for ADV infection cannot be completely excluded. 

## 4. Materials and Methods

### 4.1. Study Area and Sampling 

Muscle exudates were recovered from wild boars (n = 399) caught during two hunting seasons (season 2019–20; n = 253 and season 2021–22; n = 146) from three military camps located at Bouches-du-Rhône and Var in southeastern France [Canjuers (43°39′59.7″–6°27′54.7″), n = 306, and Carpiagne (43°14′50.5″–5°31′34.1″), n = 55, respectively] and Haute-Corse [Solenzara (41°55′46.3″–9°24′49.3″), n = 38] (Figure S1). The department of Haute-Corse is located in Corsica, a French Mediterranean island which is not free of AD in pig herds. Wild boars are the main wild large mammalian species in the three areas. As no live animal was used in the present study, approval from the ethics committee was not required; all samples used were provided by hunters with appropriate wild boar hunting licenses. 

### 4.2. Anti-gB Antibodies Detection

For the detection of the anti-gB antibodies directed against ADV, we screened muscle fluids as described elsewhere [20,21,46]. Briefly, a muscular piece of around 10 g was sampled from the costal part of the diaphragm of each animal and was lacerated and placed in a hermetic plastic bag before being frozen at −30 °C until use. The muscle fluids were obtained after gentle thawing for two hours at ambient temperature, followed by overnight incubation at 4 °C. Harvested muscle fluids were then ELISA-screened using the commercial ID Screen® Aujeszky gB Competition kit (Innovative Diagnostics, Grabels, France). The assay targets the glycoprotein B (gB) of the ADV. All muscle fluids were assayed according to the manufacturer’s instructions given for sera. Samples were considered positive if the percentage of competition (S/P%) was less than or equal to 30%, and negative if clearly greater than 40%. Between 30% and 40%, samples were classified as doubtful.

### 4.3. Statistical Analyses

For comparison between groups, only confirmed positive samples were considered for the calculation of seroprevalences. The exact binomial 95% confidence interval was used to calculate the seroprevalence of AD. The individual weight of each animal was used to predict its age according to the growth curve of European wild boars [47]. Binary logistic regression using the Newton–Raphson algorithm was used to analyze the differences in ADV seroprevalence in wild boar according to geographical location, gender, age range and sampling time. A significant effect was considered at *p*-value ≤ 0.05. All statistical analyses were performed using Addinsoft 2018 (XLSTAT 2018: Data Analysis and Statistical Solution for Microsoft Excel, Paris, France) [48].

## 5. Conclusions

In France, AD is one of the regulated animal diseases of national interest (Ministerial Order of 3 May 2022) [43]. The alert system is based on event monitoring supplemented by clinical monitoring. Apart from the few canine cases of AD declared, France remains officially free of AD after measures to eradicate the infection in pig farming (recent cases from 2018 and 2019) [16,23]. Despite the declared AD-free status of pig farms in France, the present study highlights the epidemiological pressure caused by ADV circulating in wild boar and the challenge this represents for pig farms, carnivores, and cattle in these areas. Therefore, continuous epidemiological surveillance, as well as identification of strains circulating in the wild boar population, are necessary to prevent the spread of this disease among domestic animals (Ministerial Order of 16 October 2018) [29].

## Figures and Tables

**Table 1:** Comparative analysis of AD seroprevalence according to the population demography of wild boar from Southeastern France.

Demography			ELISA Results		Statistics		
Period	Parameters	Tested	Positive	Seroprevalence (%)	Binomial 95% CI	Wald’ chi²	*p*-Value
2019–20	Sites						
	Canjuers	219	64	29.2	[23.3; 35.7]		
	Carpiagne	20	0	0		6.65	0.01
	Solenzara	13	10	76.9	[46.2; 94.9]	70.50	<0.0001
	Gender						
	Female	166	48	28.9	[22.2; 36.5]		
	Male	86	26	30.2	[20.8; 41.1]	1.23	0.267
	Age (Weeks)						
	[≤20]	61	13	21.3	[11.9; 33.7]		
	[20–35]	180	57	31.7	[25.0; 39.0]	6.16	0.013
	[35–45]	11	4	36.4	[10.9; 69.2]	19.76	<0.0001
	[≥45]	0					
2021–22	Sites						
	Canjuers	87	37	42.5	[32.0; 53.6]		
	Carpiagne	35	0	0		11.79	0.0001
	Solenzara	25	10	40	[21.1; 61.3]	3.05	0.081
	Gender						
	Female	81	26	32.1	[22.2; 43.4]		
	Male	66	21	31.8	[20.9; 44.4]	1.72	0.190
	Age (Weeks)						
	[≤20]	27	7	25.9	[11.1; 46.3]		
	[20–35]	55	13	23.6	[13.2; 37.0]	4.36	0.037
	[35–45]	46	17	37.0	[23.2; 52.5]	12.48	0.0004
	[≥45]	19	10	52.6	[28.9; 75.6]	11.85	0.0005

## Data Availability

The data presented in this study are available in the main text.

## Supplementary Materials

The following are available online at https://www.mdpi.com/article/10.3390/pathogens11101107/s1, Figure S1: Geographical map showing the location of the investigated areas: 1. Carpiagne, 2. Canjuers and 3. Solenzara.
