# Supplementary material for: Serological Survey of Aujeszky’s Disease in Wild Boar from Southeastern France
Source: Pathogens. 2022 Sep 27;11(10):1107. doi: 10.3390/pathogens11101107 (PMC9610145; doi:10.3390/pathogens11101107)
Supplement: Supplementary file 1 [file pathogens-11-01107-s001.zip › pathogens-1902924-supplementary.pdf]

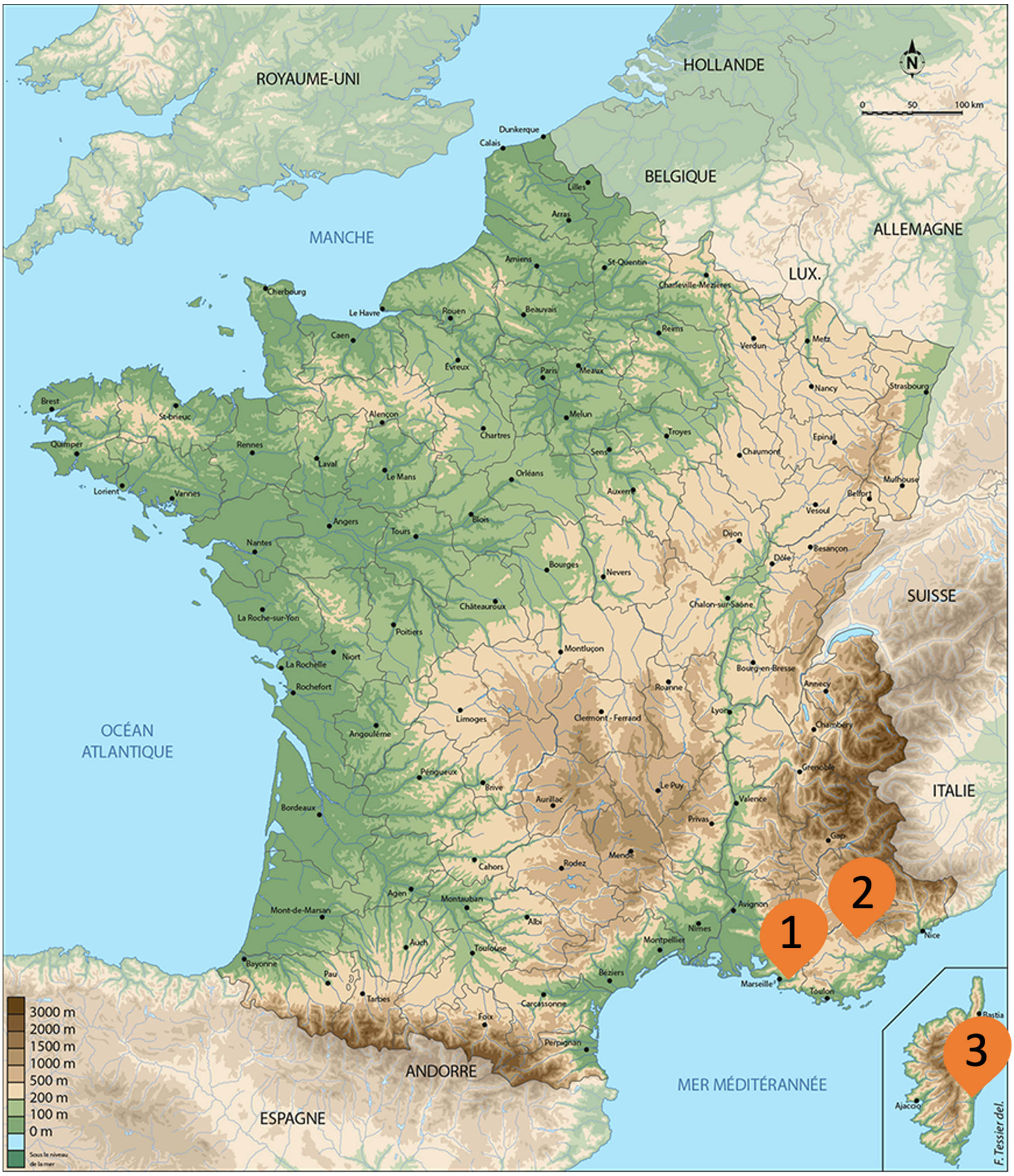

Figure S1. Geographical map showing the location of the investigated areas: 1. Carpiagne, 2. Canjuers and 3. Solenzara.
